# The Impact of an Engagement Solution Incorporating Antihypertensive Medications, Engagement Tools, and a Digital Application on Adherence Among Hypertensive Patients: A Real-World Study

**DOI:** 10.7759/cureus.107606

**Published:** 2026-04-23

**Authors:** Erika M Goncalves Campana, Rodrigo J De Castro Sepetiba, Felipe R Pacheco De Souza, Gustavo Mantovani Pucci, Paulo H Gasques Trovati, Sayuri Inuzuka, Paulo C Ferraz Dias Filho, Marselha Marques Barral Montessi, Vitor B Teixeira de Holanda, Bruno A Alcova Nogueira, Lays Paulino Leonel, Renato De Lima Vitorasso, Marcella Chaves Flores, Mayara L Da Silva

**Affiliations:** 1 Department of Cardiology, Universidade do Estado do Rio de Janeiro, Niterói, BRA; 2 Department of Cardiology, Universidade Federal de Santa Catarina ( UFSC), Santa Catarina, BRA; 3 Department of Cardiology, Policlínica Metropolitana do Estado do Pará, Belém, BRA; 4 Department of Cardiology, Cor e Ar Centro Cardio Respiratório, São Paulo, BRA; 5 Department of Cardiology, Clínica Santoro (Itu-SP), São Paulo, BRA; 6 Department of Cardiology, Universidade Federal de Goiás, Goiana, BRA; 7 Department of Cardiology, Hospital São Domingos, São Luis, BRA; 8 Department of Cardiology, Faculdade de Ciências Médicas e da Saúde de Juiz de Fora, Juiz de Fora, BRA; 9 Department of Cardiology, Universidade Estadual do Pará, Belém, BRA; 10 Department of Cardiology, Clínica Coração Vivo, São José dos Campos, BRA; 11 Department of Data Analysis, IQVIA Solutions Brazil, São Paulo, BRA; 12 Department of Medical Affairs, Servier Brazil, Rio de Janeiro, BRA

**Keywords:** database management systems, digital health, hypertension, medication adherence, patient-centered care

## Abstract

Background: Hypertension is the leading preventable risk factor for cardiovascular disease. The lack of treatment adherence is one of the most important challenges in managing and controlling hypertension. It is unclear whether patient-focused strategies and training for doctors are associated with better adherence and control.

Objective: This study aimed to evaluate treatment adherence among hypertensive patients by comparing a group of patients who were part of a patient support program (PSP) and were using the engagement solution (ES), i.e., antihypertensive medications, engagement tools, and the Elfie application. These patients were included in group A by trained physicians. Group B features patients who were also part of the program but were using only medications.

Methods: This observational and retrospective study analyzed data from the PSP Sempre Cuidando (Laboratórios Servier do Brasil, Rio de Janeiro, BRA) and the Elfie app (Elfie, SGP), collected from across Brazil between November 2022 and October 2023. The population included hypertensive patients aged 18 years or older with arterial hypertension and possessing a medical prescription for antihypertensives referenced in the PSP.

Results and conclusion: Patients were divided into two groups: 492 in group A and 984 in group B. The two groups were followed for six months. Group A demonstrated higher adherence to treatment, with an average treatment duration of 3.7 months compared to 2.7 months in group B, representing a 37% increase. Additionally, patients in group A showed statistically significant reductions in systolic blood pressure (SBP) and diastolic blood pressure (DBP) after six months versus their baseline.

Conclusions: The engagement solution was associated with higher levels of adherence and improved blood pressure control among hypertensive patients.

## Introduction

Hypertension is the leading preventable risk factor for cardiovascular (CV) disease and mortality worldwide [1]. It is an asymptomatic condition that progresses with structural and/or functional changes in target organs, such as the heart, brain, and kidneys [1,2]. Blood pressure (BP) control rates are low in Brazil and globally, averaging around 30%. Hypertensive patients who are treated but not controlled maintain a high risk of CV events, especially untreated individuals. One of the main reasons for the lack of hypertension control is poor adherence to treatment [2]. The 2025 Brazilian Guideline of Hypertension includes a chapter on treatment adherence and outlines key strategies to enhance it. According to this guideline, the most evidence-based and feasible strategies include self-measurement of BP, more convenient regimens, and multidisciplinary team care [2].

Real-world evidence suggests that BP self-monitoring using validated devices, along with recording measurements in digital solutions, is an effective intervention for achieving better BP control in hypertensive individuals [3]. Furthermore, digital solutions that assist patients in monitoring their health can provide valuable information for patients and healthcare providers [3]. Another pillar for improving patient care and achieving control is medical education, which enables physicians to enhance their competencies and ensure high-quality care [4].

The engagement solution was developed by Laboratórios Servier do Brasil (Rio de Janeiro, BRA) to address the needs of both physicians and patients and offers comprehensive solutions with and beyond the pills, structured around three key pillars. This program is part of an evolving landscape of digital and adherence support interventions designed to complement traditional pharmacological treatments in chronic disease management. The pillars include antihypertensive medications that are part of the Sempre Cuidando Patient Support Program (PSP), namely Acertil®, Acertalix®, Acertanlo®, and Triplixam®; engagement tools, which include patient education through emails, SMS, and WhatsApp; support from a multidisciplinary team and optional services for Sempre Cuidando members; and the Elfie application (Elfie, SGP), a free digital solution for managing cardiometabolic disease that uses gamification to encourage users to monitor their health.

The objective of this study was to evaluate treatment adherence by comparing a group of patients enrolled in the Sempre Cuidando program who used the engagement solution (antihypertensive medications, engagement tools, and the Elfie app) and were managed by trained physicians, with a group of patients in the same program who used only medications. For methodological clarity, it should be noted that group allocation was dependent on physician training and the utilization of engagement tools, a factor that may introduce baseline differences between the study cohorts [5,6].

## Materials and methods

Study setting and data source

This is an observational and retrospective study, with comparative analysis, using data from the Laboratórios Servier do Brasil PSP called Sempre Cuidando, collected from November 2022 to October 2023, along with data from the Elfie app. Servier and Elfie are completely independent companies. The partnership between the companies aims to assist in the adherence and continuation of treatment, without interfering with the physician's prescription. The Elfie app is owned by Elfie and is free for all patients, regardless of whether they are enrolled in Sempre Cuidando or not. Neither the companies nor the researchers had access to the patients' data.

Hypertensive patients receiving treatment with anti-hypertensive medications, Acertil®, Acertalix®, Acertanlo®, or Triplixam®, were eligible to enroll in Sempre Cuidando. It is important to note that the medical professional is responsible for diagnosing high BP and selecting the appropriate antihypertensive medication for treatment. If any of the abovementioned antihypertensive medications are prescribed, the patient is then advised to register for the Sempre Cuidando program. However, participation in the program remains entirely at the patient's discretion.

**Figure 1:**
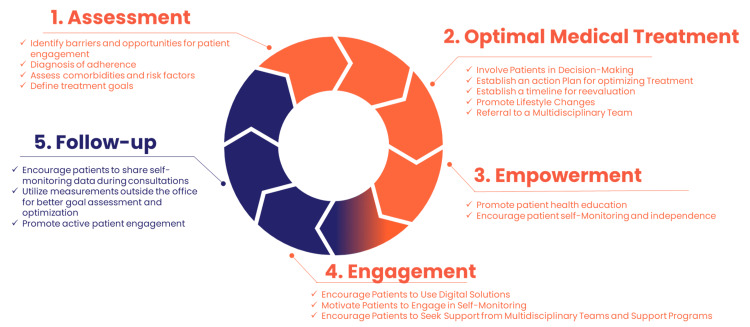
The CARE methodology CARE: Comprehensive approach to rehabilitation methodology [2,7] Diagram created by authors using Microsoft Powerpoint (Microsoft Corp., Redmond, WA, USA)

In addition to the patient-focused engagement solution described above, physician-focused interventions are essential. Therefore, the inclusion of patients in group A was done by physicians who had been trained following the comprehensive approach to rehabilitation (CARE) methodology, a medical education activity developed by Laboratórios Servier do Brasil that integrated knowledge in hard skills, which are more scientific and technical; soft skills, which are related to behavior, emotional aspects, and social interaction with patients; and digital skills on patient management through technology. This training focused on implementing a five-step methodology for better patient care: thorough assessment of the patient's condition, implementation of optimized medical therapy, patient empowerment and engagement, and patient follow-up through digital solutions, such as Elfie. The goal of this training was to transform patient care, increase adherence, and improve health engagement (Figure 1).

Population

The inclusion criteria are as follows: patients aged 18 years or older with hypertension, who have a medical prescription for the antihypertensives referenced in the Sempre Cuidando program, and whose physician possesses a valid medical professional record. Additionally, patients must agree to participate in the program.

Patients were stratified into two groups, with a 1:2 ratio, respectively. Group A included patients who were treated by a trained physician with the CARE methodology (in which hypertension-management training and access to the engagement tools are provided as a single, linked package); prescribed any of the antihypertensive medications Acertil®, Acertalix®, Acertanlo®, or Triplixam®; and decided to participate in the Sempre Cuidando program, utilizing the engagement solution (antihypertensive medications, engagement tools, and Elfie application).

Group B featured patients whose hypertension was diagnosed by a non-trained physician, prescribed any of the antihypertensive medications Acertil®, Acertalix®, Acertanlo®, or Triplixam®, and decided to participate in the Sempre Cuidando program, but were only using the medication and not utilizing the engagement tools and Elfie app. Patients who chose to withdraw from the Sempre Cuidando program and/or the Elfie app were excluded from the study.

Data collection

An anonymized database was extracted from the Sempre Cuidando program. The Elfie app also provides a database with self-reported data from the patients, which was also analyzed in this study. Patients were followed for at least six months, and the data were treated for plausibility. The relevant data time points for group A are presented in the data collection schedule provided in Table 1. All data elements were prospectively recorded from the databases of the Sempre Cuidando program and the Elfie app.

**Table 1:** Data collection schedule for group A Type of data sourced from the Sempre Cuidando program and the Elfie app database.

Data Elements	Baseline	End of follow-up (six months later for each patient)
		+/- 30 weeks
Demography	X	
Blood pressure records (self-reported by the patient) for patients using the Elfie app	X	X
HbA1c, lipid parameters, weight, and steps (self-reported by the patient) for patients using the Elfie app	X	
Concomitant medications	X	X

The demographic and clinical profiles of patients from both groups (A and B) were assessed. The absolute and relative frequencies of the demographic profile were evaluated, including age, gender, state, and region of residence. Additionally, the medication used in the Sempre Cuidando program for hypertension management was analyzed. The comparison of treatment adherence between Groups A and B was conducted in three steps.

Monthly Adherence

This was measured by dividing the total number of medicine boxes dispensed in six months by the total number of patients who enrolled in that month. The reference month was the first month in which the patient retrieved the medication. This approach provides an average monthly adherence calculation. The adherence per month was calculated as follows:

$Adherence \ [entry \ month]=\frac{Total \ number \ of \ medicine \ boxes \ dispensed \ in \ six \ months \ [entry \ month]}{Total \ number \ of \ patients \ [entry \ month]}$

Group Adherence

A six-month follow-up was considered for each patient. The total medical adherence for each group was calculated by taking the weighted average of monthly adherence rates, where each month's adherence was multiplied by the number of patients for that month, and then dividing the sum of these products by the total number of patients across all months. The equation is as follows:

$Total \ adherence= \frac{\sum_{nov/2022}^{oct/2023}Adherence \ [month]*patients \ [month]}{\sum_{nov/2022}^{oct/2023}patients \ [month]}$

Difference Between the Groups

After calculating the total adherence for groups A and B, a simple comparison of the totals was made to determine the difference between the groups. Certain outcomes were assessed only for patients enrolled in group A who self-reported information in the Elfie app due to its capacity for longitudinal data collection. Continuous clinical variables, such as systolic blood pressure (SBP) and diastolic blood pressure (DBP), hemoglobin A1c (HbA1c%), high density lipoprotein (HDL)-c (mg/dL), low density lipoprotein (LDL)-c (mg/dL), triglycerides (mg/dL), total cholesterol (mg/dL), and weight (kg), were analyzed using mean, standard deviation (SD), median, and minimum and maximum values. The Mann-Whitney test was used to compare SBP and DBP between groups A and B due to the non-normality characteristics of the sample (Shapiro-Wilk test).

Data analysis

Analysis of the difference in SBP and DBP between the baseline and after six months of follow-up in group A was performed only if the patients had more than five measurements recorded in the Elfie app, with a minimum of 30 days between two recorded assessments. To compare the difference between the two periods, a Wilcoxon test was performed, once more, due to the non-normality characteristics of the sample. Data processing was performed using Python version 3.7.7 (Python Software Foundation, Wilmington, DE, USA).

The study protocol was submitted to the Ethics Committee of Hospital Universitário Pedro Ernesto da Universidade do Estado do Rio de Janeiro (HUUERJ) located in Rio de Janeiro, Brazil (approval no. 6.526.184). A waiver of the informed consent form was obtained. Patients were informed at the time of registration or via e-mail/SMS that their data could be used for research purposes. This study was also fully compliant with resolution No. 510/2016 of the National Research Ethics Commission of Brazil (CONEP) and the Declaration of Helsinki, which outlines the ethical principles for medical research involving human subjects.

## Results

Baseline characteristics

A total of 1,471 participants were enrolled in the study. Group A comprised 492 patients, while group B included 984. The mean age of patients was similar between both groups: 52.63 years (SD: 12.55) for group A, and 52.66 years (SD: 16.24) for group B. In group A, men were slightly more numerous than women, with 51.02% (n = 251), while in group B, 50.81% (n = 500) of patients were female. In both groups, most participants resided in the Southeast region of Brazil, accounting for 62.80% in group A and 39.63% in group B, mainly in São Paulo (Table 2).

**Table 2:** Demographic characteristics

Variables, n (%)		Group A (n=492)	Group B (n=984)
Age (in years)	Mean (SD)	52.63 (12.55)	52.66 (16.24)
	Median (Q1; Q3)	51.87 (43.61; 61.64)	53.09 (40.13; 65.67)
	Minimum	22.33	20.34
	Maximum	92.26	92.42
	Total available information	492	984
Gender	Male	251 (51.02)	483 (49.09)
	Female	241 (48.98)	500 (50.81)
	Total available information	492	983
	Missing	0	1
State of residence	Acre	0 (0.00)	1 (0.10)
	Alagoas	0 (0.00)	14 (1.43)
	Amapá	0 (0.00)	3 (0.31)
	Amazonas	0 (0.00)	5 (0.51)
	Bahia	2 (0.41)	19 (1.94)
	Ceará	3 (0.61)	33 (3.37)
	Distrito Federal	9 (1.83)	25 (2.55)
	Espírito Santo	3 (0.61)	20 (2.04)
	Goiás	30 (6.10)	27 (2.76)
	Maranhão	18 (3.66)	26 (2.66)
	Mato Grosso	1 (0.20)	5 (0.51)
	Mato Grosso do Sul	10 (2.03)	15 (1.53)
	Minas Gerais	25 (5.08)	77 (7.87)
	Pará	78 (15.85)	20 (2.04)
	Paraíba	0 (0.00)	15 (1.53)
	Paraná	0 (0.00)	58 (5.92)
	Pernambuco	4 (0.81)	22 (2.25)
	Piauí	0 (0.00)	9 (0.92)
	Rio de Janeiro	31 (6.30)	80 (8.17)
	Rio Grande do Norte	0 (0.00)	23 (2.35)
	Rio Grande do Sul	13 (2.64)	184 (18.79)
	Rondônia	0 (0.00)	4 (0.41)
	Roraima	0 (0.00)	0 (0.00)
	Santa Catarina	9 (1.83)	68 (6.95)
	São Paulo	250 (50.81)	213 (21.76)
	Sergipe	6 (1.22)	13 (1.33)
	Tocantins	0 (0.00)	0 (0.00)
	Total available information	492	979
Region of residence	North	78 (15.85)	38 (3.86)
	Northeast	33 (6.71)	174 (17.68)
	South	22 (4.47)	310 (31.50)
	Southeast	309 (62.80)	390 (39.63)
	Central-West	50 (10.16)	72 (7.32)

The clinical characteristics assessed for group A using the Elfie app are described in Table 3. The mean SBP was 129.37 mmHg (SD: 15.09), and DBP was 80.74 mmHg (SD: 9.84). The mean HbA1c was 7.17% (SD: 1.69). The mean levels of HDL-c, LDL-c, triglycerides (TG), and total cholesterol were 50.56 mg/dL (SD: 22.01), 114.28 mg/dL (SD: 44.99), 151.96 mg/dL (SD: 106.51), and 195.22 mg/dL (SD: 60.38), respectively. The mean weight of the patients was 81.25 kg (SD: 18.65).

**Table 3:** Clinical characteristics of group A sourced from the Elfie app data SBP: Systolic blood pressure, DBP: Diastolic blood pressure, HbA1c: Hemoglobin A1c, HDL: High density lipoprotein, LDL: Low density lipoprotein, TG: Triglycerides

Parameter	Mean (SD)	Median (Q1; Q3))	Range (Min-Max)
SBP (mmHg) (n=356)	129.37 (15.09)	126.64 (119.84; 135.69))	(100.00 - 200.00)
DBP (mmHg) (n=356)	80.74 (9.84)	80.00 (75.00; 85.00))	(57.00 - 130.00)
HbA1c (%) (n=56)	7.17 (1.69)	7.35 (5.98; 7.60)	(4.60 - 13.68)
HDL-c (mg/dL) (n=93)	50.56 (22.01)	46.00 (38.00; 57.00)	(1.00 - 150.00)
LDL-c (mg/dL) (n=93)	114.28 (44.99)	114.00 (90.00; 143.00)	(1.00 - 207.00)
TG (mg/dL) (n=93)	151.96 (106.51)	120.00 (91.00; 171.00)	(1.00 - 719.00)
Cholesterol total (mg/dL) (n=93)	195.22 (60.38)	198.00 (164.00; 224.00)	(2.00 - 392.00)
Weight (Kg) (n=477)	81.25 (18.65)	80.00 (66.52; 92.80)	(27.90 - 148.27)

Medication

Triplixam® was the most prescribed medication, used by 45.9% of group A and 35.8% of group B. Acertalix® was used by 22.1% of group A and 17.7% of group B. Acertanlo® was prescribed to 20.1% of group A and 21.6% of group B (Table 4).

**Table 4:** Frequency of medication used

Medications, n (%)	Group A (n=457)	Group B (n=909)	Total (n=1,366)
Triplixam^®^	210.0 (45.9%)	325 (35.8%)	535 (39.2%)
Acertalix^®^	101.0 (22.1%)	161 (17.7%)	262 (19.2%)
Acertanlo^®^	92.0 (20.1%)	196 (21.65)	288 (21.1%)
Acertil^®^	54.0 (11.8%)	227 (25%)	281 (20.6%)

Adherence

**Figure 2:**
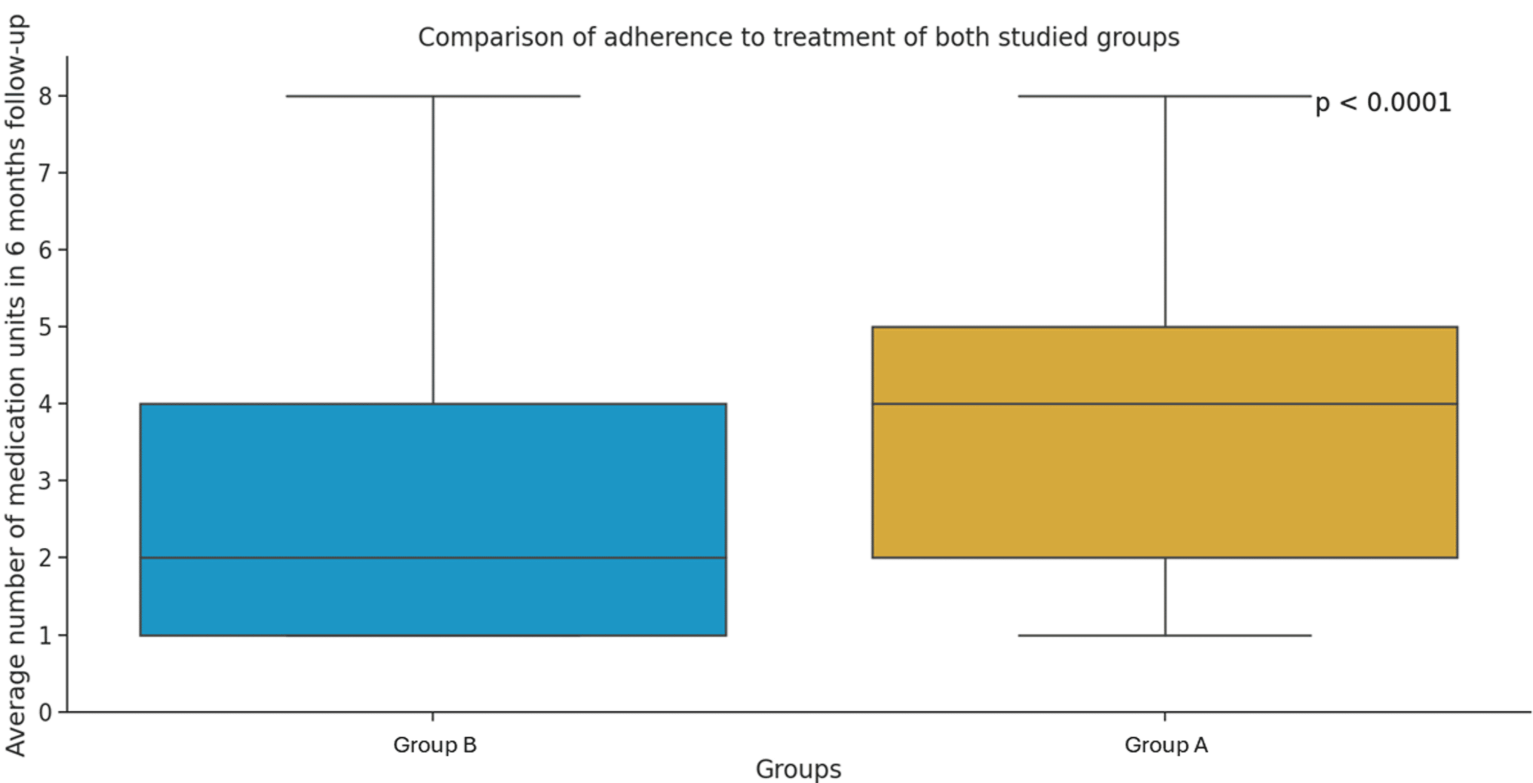
Adherence to treatment in groups A and B based on median number of medications during the six-month follow-up period The Mann-Whitney test was used to compare groups A and B.

Regarding treatment adherence, the average of total adherence was 3.77 months in group A and 2.77 months in group B. Overall, adherence was 36.1% higher in group A over the six-month period, with higher adherence observed in most months for group A (Table 5; Figure 2).

**Table 5:** Monthly and total adherence characteristics per group during the six-months of follow-up *Adherence: Medication retrieval over six months

Enrolment		Group A		Group B	
Month	n	n	Adherence*	n	Adherence*
November 2022	39	13	3.00	26	2.88
December 2022	69	23	3.78	46	3.00
January 2023	45	15	3.47	30	3.47
February 2023	54	18	3.72	36	3.25
March 2023	90	30	3.27	60	3.15
April 2023	84	28	4.11	56	2.89
May 2023	114	38	3.84	76	2.79
June 2023	156	52	4.06	104	2.78
July 2023	219	73	3.84	146	2.87
August 2023	228	76	3.76	152	2. 03
September 2023	240	80	3,.95	160	2.51
October 2023	138	46	3.46	90	3.40
Total adherence	1476	492	3.77	984	2.77

**Figure 3:**
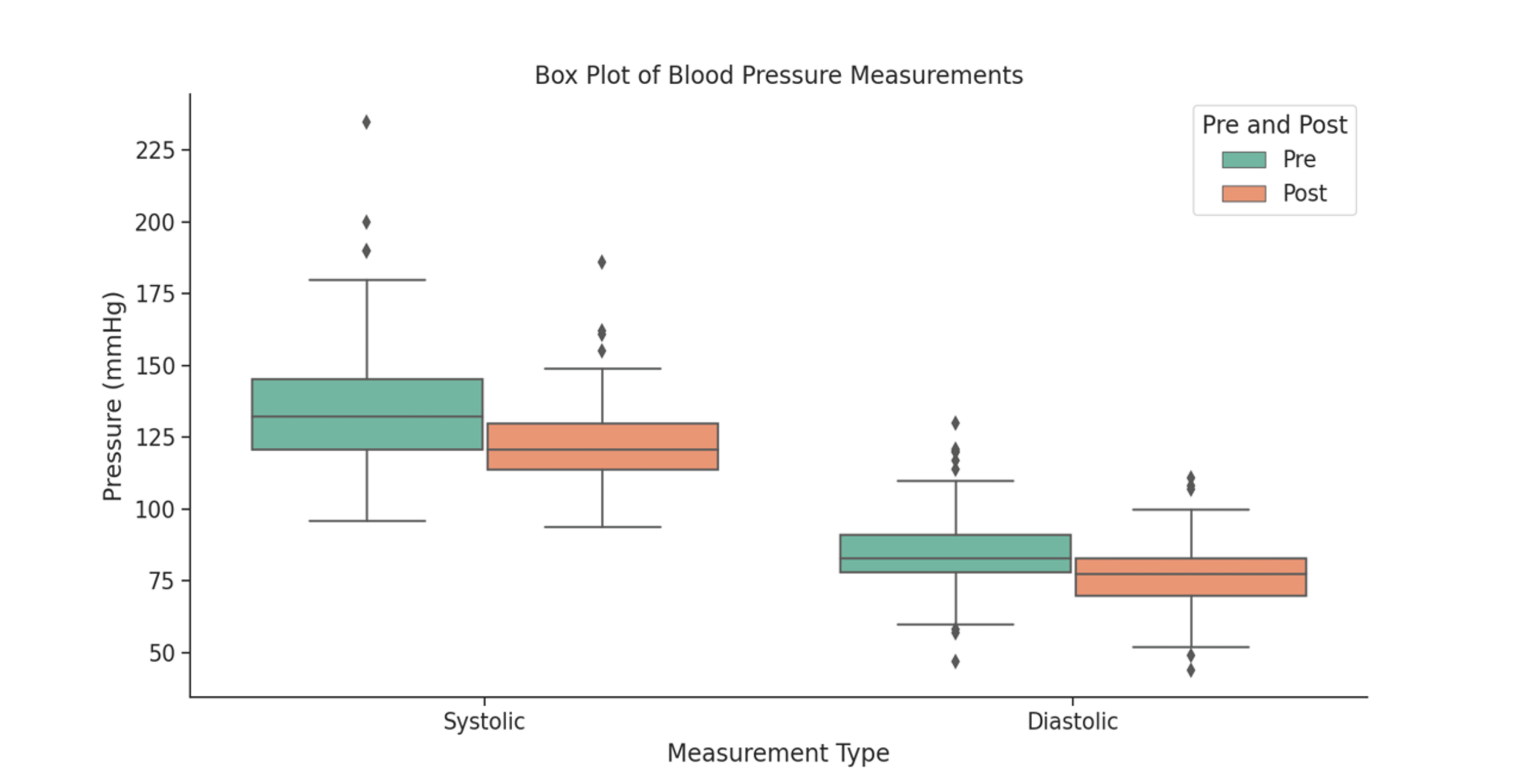
Assessment of reduction in blood pressure of group A patients

Table 6 presents the average values of SBP and DBP for group A at two different time points: baseline and sixth month (or last follow-up). There was a statistically significant difference (p <0.0001) when comparing these periods. For SBP, the mean at baseline was 135.98 mmHg (SD: 19.89), decreasing to 122.39 mmHg (SD: 12.73) after the sixth month. For DBP, the mean at baseline was 85.07 mmHg (SD: 12.85), decreasing to 76.65 mmHg (SD: 10.27) after the sixth month. Additionally, the boxplot in Figure 3 highlights the reduction in blood pressure values for group A.

**Table 6:** Assessment of reduction in blood pressure for group A patients The Wilcoxon test was used to compare baseline vs. last follow-up.

Parameter			Baseline	Last follow-up	p-value
SBP (mmHg)	Mean (SD)		135.98 (19.89)	122.39 (12.73)	< 0.0001
	Median (Q1; Q3)		132.50 (120.75; 145.50)	121.00 (113.75; 130.00)	
	Range (min-max)		(96.00-235.00)	(94.00-186.00)	
	Total available information		188	188	
DBP (mmHg)	Mean (SD)		85.07 (12.85)	76.65 (10.27)	< 0.0001
	Median [Q1; Q3]		83.00 (78.00; 91.25)	77.50 (70.00; 83.00)	
	Range (Min-Max)		(47.00 - 130.00)	(44.00 - 111.00)	
	Total available information		188	188	

## Discussion

In this study that evaluated a patient-focused engagement solution and training for healthcare professionals, medication adherence increased by 36.1% after a six-month follow-up for the group of patients who used the engagement solution, i.e., antihypertensive medication, engagement tool, and the Elfie app, compared to the group of patients who used only antihypertensive medications. To our knowledge, this is the first study providing hypertension information through a database from a PSP.

Suboptimal adherence to antihypertensive medication significantly contributes to the lack of BP control, resulting in dramatic numbers of uncontrolled patients, around 70% in Brazil. This causes hypertension to remain the leading preventable risk factor for all-cause mortality worldwide [1,2]. The consequences of non-adherence to antihypertensive medications can even increase healthcare costs [1]. Adherence to treatment is a multifaceted issue, and many barriers must be addressed at the healthcare system level. However, from a practical perspective, the doctor-patient relationship is essential for identifying adherence challenges and selecting interventions tailored to engage the individual patient [8].

Prescribing single-pill combinations (SPC) allows physicians to reduce pill burden and simplify treatment, effectively addressing two significant factors that influence adherence: polypharmacy and the complexity of treatment regimens. A meta-analysis involving approximately 18,000 patients found that the use of SPCs containing two or more antihypertensive agents was associated with a 29% increase in adherence and persistence with treatment compared to using the same two drugs in free combination [7].

At baseline, patients presented with multiple comorbidities, including diabetes, dyslipidemia, and obesity, and were not meeting the targets established by clinical guidelines. These clinical characteristics justify the frequent use of dual and triple therapy for hypertension management. Furthermore, the preference for triple therapy as a primary choice is consistent with the cardiovascular risk profile of Brazilian patients, who are often categorized as very high risk. Given Brazil's continental dimensions, there is a higher concentration of physicians and patients in the South and Southeast regions, which accounts for the greater representation of patients from these areas in the study. [2]

In the Sempre Cuidando program, patients have access to once-daily monotherapy and fixed combinations (Acertil®, Acertalix®, Acertanlo®, and Triplixam® [9-12] from Servier), which consist of perindopril, indapamide, and amlodipine. These molecules have a robust scientific background from randomized clinical trials, demonstrating not only blood pressure control but also a decrease in major cardiovascular events, including mortality [2,9-12].

Additionally, electronic health tools have shown promise in enhancing adherence. These strategies have the potential to improve cardiovascular outcomes and reduce healthcare costs [7]. In a large cohort of individuals with elevated BP or hypertension, participation in a self-management program that included a BP monitor and a connected smartphone app with clinically based automated lifestyle coaching was associated with lower BP over a follow-up period of up to three years [3].

The engagement solution was developed aiming to increase patients' access to exactly the strategies mentioned above: anti-hypertensive medication as an SPC; an engagement tool through educational content via SMS, e-mail, and WhatsApp; multidisciplinary team support; and the Elfie app. Since adherence is a complex, multidimensional process influenced by a wide range of factors, the use of applications such as Elfie, which include gamification and personalized health plans, has been a strategy increasingly used by patients and recommended by physicians to improve accessibility, adherence, and engagement of patients in therapy and lowering blood pressure as patients become more involved in their care [3].

In this study, the combined intervention, encompassing physician training, engagement tools, and use of the Elfie app, appeared to enhance patient engagement and support more consistent medication use. Because these components were delivered together, their individual contributions cannot be separated. Among patients exposed to the full engagement solution and with available Elfie app data, a statistically significant reduction in SBP and DBP was observed after six months of follow‑up, with mean decreases of approximately 13 mmHg and 9 mmHg, respectively. This reinforces the idea that support from healthcare professionals and digital interventions can lead to improved disease outcomes, likely due to continuous monitoring and real-time feedback that encourages patients to adhere to treatment and implement lifestyle changes, consistent with published studies [2,3,7].

The main limitations of this study relate to potential reporting bias. The quality of the data depends on the integrity and consistency of the records in the Sempre Cuidando program database and in the Elfie app. In addition, information collected through the app is self‑reported, which may affect its accuracy and reliability. Because patient group allocation was linked to physicians’ prior participation in the training component of the program, some degree of selection bias or influence of provider characteristics may be present, although the extent of this effect cannot be fully assessed. Finally, the adherence measure reflects medication dispensing or retrieval rather than confirmed medication intake and, therefore, may overestimate true adherence.

The adherence evaluated in this study reflects information on the availability of antihypertensive drugs from Servier in Brazilian pharmacies, through the registration of these patients. However, we cannot ensure that all patients who purchase antihypertensive medications from Servier are registered in the program or that the patient purchased the medication outside the program. Additionally, just because a drug is reported as purchased does not guarantee that it will be taken as prescribed.

## Conclusions

This study suggests that implementing engagement strategies for patients and physicians may be associated with improved treatment‑related behaviors. The findings indicate that patients who engage with the full engagement solution (antihypertensive medications, engagement tools, and the Elfie app) and are managed by physicians trained in the program tend to show higher treatment adherence and are associated with better blood pressure control. Overall, these strategies appear to be promising approaches that may support the management of chronic conditions such as hypertension.

## References

[1] WORLD HEALTH ORGANIZATION (2025). Global report on hypertension: the race against a silent killer. World Health Organization.

[2] Brandão AA, Rodrigues CI, Bortolotto LA (2025). Brazilian Guidelines of Hypertension - 2025. Arq Bras Cardiol.

[3] Gazit T, Gutman M, Beatty AL (2021). Assessment of hypertension control among adults participating in a mobile technology blood pressure self-management program. JAMA Netw Open.

[4] Lim SC, Mustapha FI, Aagaard-Hansen J, Calopietro M, Aris T, Bjerre-Christensen U (2020). Impact of continuing medical education for primary healthcare providers in Malaysia on diabetes knowledge, attitudes, skills and clinical practices. Med Educ Online.

[5] (2025). Programa Sempre Cuidando. https://semprecuidando.com.br/.

[6] (2025). Elfie • digital health • it pays to get better. https://www.elfie.co/.

[7] Mancia G, Kreutz R, Brunström M (2023). 2023 ESH Guidelines for the management of arterial hypertension The Task Force for the management of arterial hypertension of the European Society of Hypertension: endorsed by the International Society of Hypertension (ISH) and the European Renal Association (ERA). J Hypertens.

[8] Poulter NR, Borghi C, Parati G, Pathak A, Toli D, Williams B, Schmieder RE (2020). Medication adherence in hypertension. J Hypertens.

[9] Servier. Bula Profissional da Saúde (2025). Bula Profissional da Saúde - Acertalix 5/1,25 mg. https://servier.com.br/wp-content/uploads/sites/42/2026/04/Acertalix_5mg_Profissional-de-saude.pdf.

[10] Servier. Bula Paciente (2025). Bula Paciente - Acertanlo® 3,5mg/2,5mg; 7mg/5mg; 14mg/10mg. https://servier.com.br/wp-content/uploads/2024/07/ACERTANLO-Bulas-para-o-paciente.pdf.2024..

[11] Servier. Bula Profissional da Saúde (2025). Bula Profissional da Saúde - Triplixam. https://servier.com.br/wp-content/uploads/2024/04/24.04.03_BU_PS_TRIPLIXAM_.pdf.2024..

[12] Servier. Bula Profissional da Saúde (2025). Bula Profissional da Saúde - Acertil. https://servier.com.br/wp-content/uploads/sites/42/2026/01/acertil-ps-br.pdf.

